# The Combination of MBP and BCG-Induced Dendritic Cell Maturation through TLR2/TLR4 Promotes Th1 Activation In Vitro and Vivo

**DOI:** 10.1155/2017/1953680

**Published:** 2017-02-15

**Authors:** LiNa Jiang, GuoMu Liu, WeiHua Ni, NanNan Zhang, Jing Jie, Fei Xie, GuiXiang Tai

**Affiliations:** Department of Immunology, College of Basic Medical Science, Jilin University, Changchun 130021, China

## Abstract

To explore whether TLR2/TLR4 could be involved in the maturation of dendritic cells and polarization of CD4^+^ T cells induced by dendritic cells stimulated with MBP and BCG, in vitro and in vivo experiments using TLR2^−/−^ or TLR4^−/−^ mice were employed. MBP and BCG elevated CD80, CD86 and MHC class II expressed on dendritic cells and increased IL-12 protein, induced DC maturation, and indirectly promoted Th1 activation. Moreover, MBP and BCG upregulated costimulatory molecules on DCs in a TLR2- and TLR4-dependent manner. The levels of IFN-*γ*, IL-4, and IL-10 in CD4^+^ T cells cocultured with dendritic cells from different types of mice were determined with ELISPOT or ELISA method. TLR2/TLR4 is important in the maturation and activation of dendritic cells and the activation of Th1 cells induced by stimulation with MBP and BCG. In conclusion, TLR2 and TLR4 play an important role in the upregulation of costimulatory molecules and MHC class II molecules on dendritic cells and the activation of Th1 cells induced by stimulation with MBP and BCG. The results above indicate that the combination of MBP and BCG induced the maturation and activation of dendritic cells and promoted Th1 activation via TLR2/TLR4.

## 1. Introduction 

Various microbial components induce the activation of DCs (dendritic cells) through TLR (Toll-like receptor) signaling, which plays a critical role in the generation of protective immune responses [1]. DCs are generally viewed as the most potent professional APCs (antigen presenting cells). Depending on the morphological, phenotypic, and functional characteristics, DCs exist in the following two basic functional states: immature DCs and mature DCs [2]. Mature DCs are not only characterized as having the function of immunogenic DCs, which induce immunity to foreign antigens, but also characterized by having the phenotypic profile of DCs, which express high levels of costimulatory, adhesion and major histocompatibility complex (MHC) molecules [3]. Evidence has suggested that a marked increase in the levels of the cell surface markers indicates the maturation of DCs [4]. DC-based immunotherapeutic strategies have always played a critical role in linking DCs and T lymphocytes, which are regulated by the upregulation of costimulatory and MHC molecules as well as Toll-like receptors on DCs.

MBP (*Escherichia coli* maltose-binding protein) encoded by the malE gene is responsible for the capture and transport of maltodextrins in* E. coli (Escherichia coli)* [5]. Previously, MBP was deemed to have minimum effects on bioactivity and is frequently used as a protein tag. Recent studies have characterized the immunological attributes of MBP. It was found that MBP not only induces DC activation but also has TLR4 agonist-like properties and the ability to activate the NF-*κ*B signaling pathway [6]. Our previous experiments indicated that MBP induced the activation of Th1 cells, NK cells, and macrophages, indicating that MBP has potent immune-enhancing activities. Furthermore, we found that MUC1 and BCG show better antitumor effects after being fused to MBP and the combination of MBP and BCG can induce Th1 activation synergistically and significantly increase the IFN-*γ* production of lymphocytes [7].

TLRs are mainly expressed in immune cells and recognize microbial products to trigger innate immune responses [8, 9]. Additionally, TLRs are the most widely studied family of PRRs (pattern recognition receptors) on professional phagocytes such as macrophages and DCs [10, 11]. Other studies found that MBP directly induced macrophage activation and M1 polarization through the TLR2 and TLR4 signaling pathways [12, 13]. Our latest studies showed that Th1 polarization and TLR2/TLR4/TLR9 activation were synergistically induced by the combination of MBP and BCG and were the first to reveal that the cross-talk between TLR signaling pathways was associated with the activation of Th1 cells by the combination of MBP and BCG [14]. However, very little is known about the function and maturation of DCs that are induced by the combined effects of MBP and BCG and promote Th1 type immunity. To clarify the molecular mechanism of MBP or the combination of MBP and BCG and its potential use as a TLR2/TLR4 agonist in DC-based immune therapies, we mainly investigated the synergistic effect of the combination of MBP and BCG on the maturation and function of DCs. Furthermore, our findings highlight MBP as a TLR2/TLR4 agonist that favors DC- induced Th1 polarization indirectly.

## 2. Materials and Methods

### 2.1. Animals

C57BL/6J TLR2 knockout mice (TLR2^−/−^; B6,129 Tlr2tmikir/J) and C57BL/10 TLR4 knockout mice (TLR4^−/−^; C57BL/10SCNJ) were purchased from Model Animal research center of NanJing University. And age and sex-matched C57BL/6 Wild type (WT) mice were purchased from Laboratory Animal Center of Chinese Academy of Medical Sciences. All animals were bred and maintained under specific pathogen-free environment. And all animal studies were conducted in accordance with National Institutes of guidelines for animal care and use of laboratory animals.

### 2.2. Reagents and Antibodies

MBP was produced from an* E. coli* strain that carries the MBP expression vector pMAL-c2 (New England Biolabs, Beverly, Massachusetts, USA). The expression vector consists of MBP preceded by methionine, with the final four amino acids replaced by 23 residues encoded by the pMAL-c2 polylinker. The MBP protein was purified with affinity chromatography on amylose resin, as described in previous reports [7]. Using a polymyxin B-agarose column (Sigma-Aldrich, Saint Louis, MO, USA), the endotoxin in the MBP protein was removed using ultrafiltration techniques with Amicon Ultra-15 Centrifugal Filter Units plus Ultracel-10 (Merck Millipore, Billerica, MA, USA). The residual endotoxin level in the MBP protein was examined with a limulus amebocyte lysate-based kit (BioWhittaker, Atlanta, GA, USA) [12, 13]. The level of endotoxin in the MBP protein prepared for the experiments was less than 0.05 EU/mL.

CD11C^+^ (N418) microBeads and CD4^+^T cell isolation kit were purchased from Miltenyi Biotec GmbH, Germany. FITC-conjugated anti-CD80, PE-conjugated anti-CD86, and APC-conjugated anti-MHC class II were purchased from Miltenyi Biotec GmbH, Germany. FITC-conjugated anti-TLR2 and PE-conjugated anti-TLR4 were purchased from Biolegend (San Diego, CA, USA). Cytokine ELISPOT kits for murine IFN-*γ* and IL-4 were purchased from Mabtech, AB, Inc, Sweden. ConA reagents and CCK8 kits were purchased from Sigma.

### 2.3. The Experiment Design In Vitro

Regarding in vitro experiments, the pure DCs from normal mice or TLR2^−/−^ mice or TLR4^−/−^ mice were divided into four groups by addition of different reagents: Blank control, MBP (10 *μ*g/mL), BCG (20 *μ*g/mL), and MBP (10 *μ*g/mL) + BCG (20 *μ*g/mL). When DCs incubated with different stimulation reagents for 48 h, DCs in different groups were collected and prepared to use. One part of DCs from different groups was used to detect molecular expression on the surface of DCs, such as CD80, CD86, MHC class II, TLR2, and TLR4. The other part of DCs were cocultured with CD4^+^T cells isolated from normal mice.

### 2.4. Isolation of Dendritic Cells and CD4^+^ T Cells

The spleen samples were taken out from C57BL/6 mice or TLR4^−/−^ or TLR2^−/−^ mice, respectively, and the single-cell suspensions were prepared by enzymatic disaggregation with Collagenase D. The mononuclear cells were isolated from spleen with mouse percoll and were centrifuged by the method of ficoll density gradient. Then one part of mononuclear cells was prepared to isolate the dendritic cell after washing with PBS, the other parts of mononuclear cells were prepared to isolate CD4^+^T cells. The mononuclear cells were added with anti-CD11C microBeads and were incubated for 15 minutes at 4°C. The cells were washed by buffer and were centrifuged by 200*g* for 10 minutes. The supernatant was aspirate, and the cell suspension magnetically labeled with specific anti-CD11C microBeads is loaded onto a MACS column using positive selection, which is placed in the magnetic field of a MACS separator. The procedure of isolation of CD4^+^T cells from spleens was performed using immune-MACS as previously described [15]. Then the purities of dendritic cells and CD4^+^T cells were analyzed by flow cytometry, respectively.

### 2.5. Flow Cytometry

One part of DCs collected from different groups, cultured in a 96 well plate at a density of 2 × 10^5^ cells/well, was stained with FITC-conjugated anti-CD80, PE-conjugated anti-CD86, and APC-conjugated anti-MHC class II antibodies simultaneously. According to the above method, the other part of DCs collected from different groups was stained with FITC-conjugated anti-TLR2 and PE-conjugated anti-TLR4, respectively. Incubate for 30 min at 4°C in a fridge. For best results, analyze the cells on the flow cytometer (BD FACSVerse) as soon as possible.

### 2.6. ELISPOT

Levels of IFN-*γ* production and IL-4 production in supernatant of CD4^+^T cells cocultured with DC were detected with precoated ELISPOT (Enzyme-Linked Immunospot test) kit (Mabtech, AB, Inc, Sweden). The procedure was performed strictly according to the recommended instructions. The results of ELISPOT were analyzed with Immunospot instrument (S6 Universal, Cellular Technology Ltd).

### 2.7. The Immunization of Animals

WT mice (normal mice) or TLR2^−/−^ mice or TLR4^−/−^ mice were divided into four groups (three mice per group) randomly, including NS (control group), BCG group, MBP group, and the combination of MBP and BCG group. In the first immune experiment, mice were immunized by subcutaneous injection of MBP (2.5 mg/kg), BCG (150 mg/kg), and a combination of MBP (2.5 mg/kg) and BCG (150 mg/kg) into the back of mice. And mice were immunized subcutaneous with normal saline (NS) as a control. The second immunization began two weeks after the first immunization. On the seventh day after the second immunization, all mice were killed and spleens from each mouse were harvested to prepare for next experiments.

### 2.8. Statistical Analysis

The experiments in this study were repeated for three times. And all data were expressed as the mean ± SEM (standard error of the mean). The statistical significance for comparison between samples was determined by one-way ANOVA using SPSS software 16.0. *P* values <0.05 were considered statistically significant.

## 3. Results

### 3.1. MBP and BCG Synergistically Induced Th1 Activation In Vitro

**Figure 1:**
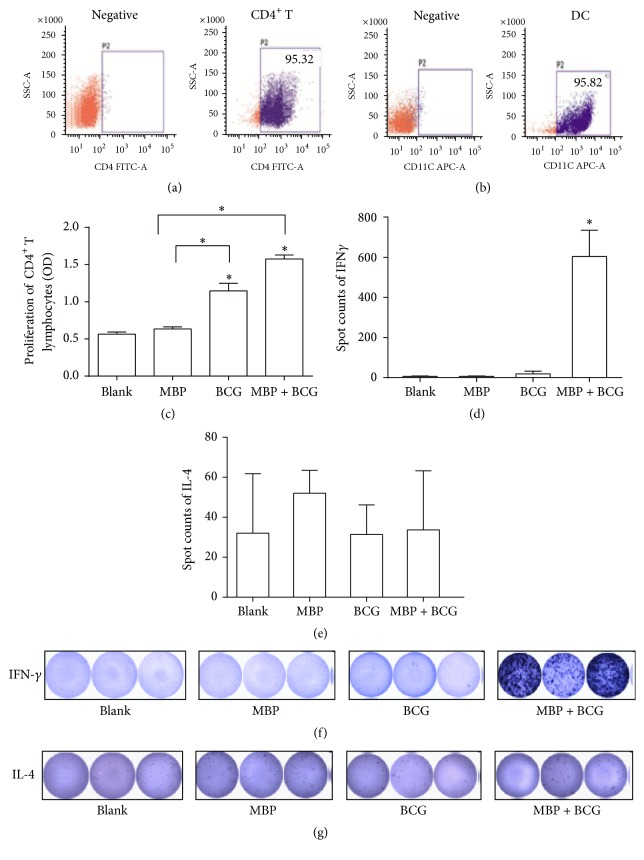
The activation of CD4^+^ T cells induced by the combination of MBP and BCG. (a) Isolation of CD4^+^ T cells from spleen samples from normal mice. The CD4^+^T cell purity was analyzed by flow cytometry. (b) Dendritic cells were isolated from the spleen, and the percentage of dendritic cells purified was measured by FACS analysis. (c) MBP and BCG synergistically increased the proliferation of CD4^+^ T cells cocultured with DCs. All experiments were repeated three times, and all the data are expressed as the mean ± SD (*n* = 3). ^*∗*^*P* < 0.05 was considered significant, compared with Blank group or with MBP group. (d and e) The production of IFN-*γ* and IL-4 in CD4^+^ T cells cocultured with DCs, as detected by ELISPOT. CD4^+^ T cells were cocultured with DCs at a ratio of 100 : 1. (f and g) Spots were counted by an ELISPOT reader.

To investigate whether DCs treated with the combination of MBP and BCG affect the proliferation of CD4^+^ T cells, the proliferation of pure CD4^+^ T cells derived from normal mice and cocultured with DCs from normal mice was detected with CCK8 kits. The pure CD4^+^ T cells and dendritic cells used in the present study were isolated from spleen samples with micro-magnetic beads, and the purity of each sorted cell population was more than 95% (Figures 1(a) and 1(b)). CD4^+^ T cells cocultured with DCs derived from WT mice were incubated with MBP (10 *μ*g/mL), BCG (20 *μ*g/mL), and the combination of MBP (10 *μ*g/mL) and BCG (20 *μ*g/mL) for 48 hours. Then, the proliferation of CD4^+^ T cells was detected using CCK8 assays. As shown in Figure 1(c), MBP, BCG, and the combination of MBP and BCG increased the proliferation of CD4^+^ T cells cocultured with DCs relative to that of the control group. Moreover, the results showed that the combination of MBP and BCG synergistically increased the proliferation of CD4^+^ T cells relative to that in the MBP group and the BCG group.

To further study the effect of the combination of MBP and BCG on the activation of CD4^+^ T cells cocultured with DCs, the secretion of IFN-*γ* and IL-4 was examined using ELISPOT. CD4^+^ T cells were cocultured with DCs stimulated with MBP, BCG, or the combination of MBP and BCG for 48 h, and the IFN-*γ* production and IL-4 production in the above groups were detected with mouse IFN-*γ* and IL-4 ELISPOT kits according to the instructions. Coculturing CD4^+^ T cells with DCs stimulated with the combination of MBP and BCG significantly increased the production of IFN-*γ* (Figures 1(c) and 1(e)) but decreased the levels of IL-4 production relative to the MBP-stimulated group and the BCG-stimulated group (Figures 1(d) and 1(f)).

### 3.2. Upregulation of Costimulatory Molecules on DCs following Stimulation with MBP, BCG, or the Combination of MBP and BCG In Vitro

**Figure 2:**
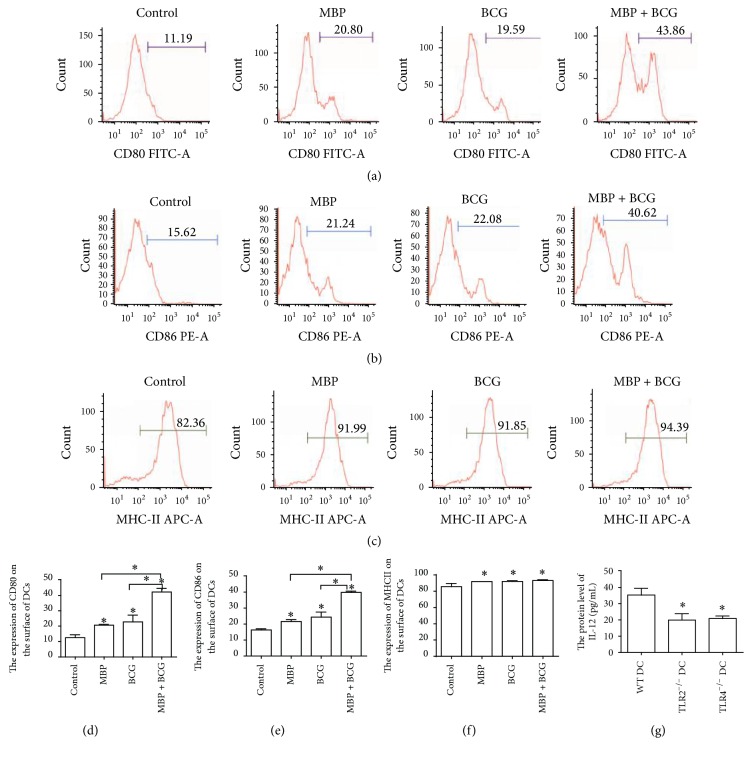
The combination of MBP and BCG upregulates CD80, CD86, and MHC class II expression on the surface of DCs and increased the production of IL-12 protein. Splenic DCs from normal mice (1 × 10^6^ cells/mL) were cultured with MBP, BCG, or the combination of MBP and BCG for 48 h and were analyzed using flow cytometry. DCs collected from the different groups were stained with the following antibodies: FITC-conjugated anti-CD80 (a), PE-conjugated anti-CD86 (b), and APC-conjugated anti-MHC class II (c). The percentage of positive cells is shown in a flow cytometry histogram. The percentage of CD11c^+^ cells expressing each surface molecule is expressed as the mean ± standard deviation and is shown in a bar graph (d–f). ELISA was used to detect the level of IL-12 protein in supernatant of DCs isolated from different types of mice (WT mice, TLR2^−/−^ mice, or TLR4^−/−^ mice) treatment with the combination of MBP and BCG (g). Data were derived from three independent experiments. ^*∗*^*P* < 0.05 is considered to indicate statistically significant differences, compared with control group or with WT DC group.

In order to determine if the combination of MBP and BCG had an effect on the activation and maturation of DCs, we characterized the expression of costimulatory molecules and MHC II molecules on DCs from normal mice following stimulation with MBP, BCG, or the combination of MBP and BCG. As shown in Figure 2, the DCs stimulated with MBP showed greater CD80 and CD86 expression than the DCs in the control group (CD80: 20.80% for MBP versus 11.19% for control; CD86: 21.24% for MBP versus 15.62% for control). Similarly, BCG increased the percentage of CD11c^+^ cells expressing CD80 and CD86 to 19.59% or 22.08%, respectively, compared to 11.59% or 15.62%, respectively, for the DCs in the control group. In particular, the combination of MBP and BCG synergistically increased the percentage of CD11c^+^ cells expressing CD80 to 43.86%, compared to 11.19% for the DCs in the control group and increased the percentage of CD11c^+^ cells expressing CD86 to 40.62%, compared to 15.62% for the DCs in the control group. In addition, MBP or BCG increased the expression of MHC class II on the surface of DCs relative to the DCs in the control group (91.99% versus 82.36%, 91.85% versus 82.36%), and the combination of MBP and BCG significantly increased the percentage of cells expressing the surface molecule MHC class II to 94.39%, compared to 82.36% of the DCs in the control group. These results demonstrated that the combination of MBP and BCG induced a more mature phenotype. Taken together, MBP and BCG synergistically induced the activation and maturation of DCs.

In addition, we detect the level of IL-12 protein in supernatant of DCs from different types of mice (WT mice, TLR2^−/−^ mice, or TLR4^−/−^ mice) treatment with the combination of MBP and BCG. As shown in Figure 2(g), the combination of MBP and BCG increased the production of IL-12 protein compared with control group (the level of IL-12 protein in control group is very low and data is not shown). However, lack of TLR2 or TLR4 in DCs decreased the protein level of IL-12 induced by the combination of MBP and BCG. It indicated that TLR2/TLR4 expressed in DCs may be associated with secretion of IL-12 protein in DCs treatment with MBP and BCG.

### 3.3. MBP Elevated TLR2/TLR4 Surface Expression on DCs In Vitro

**Figure 3:**
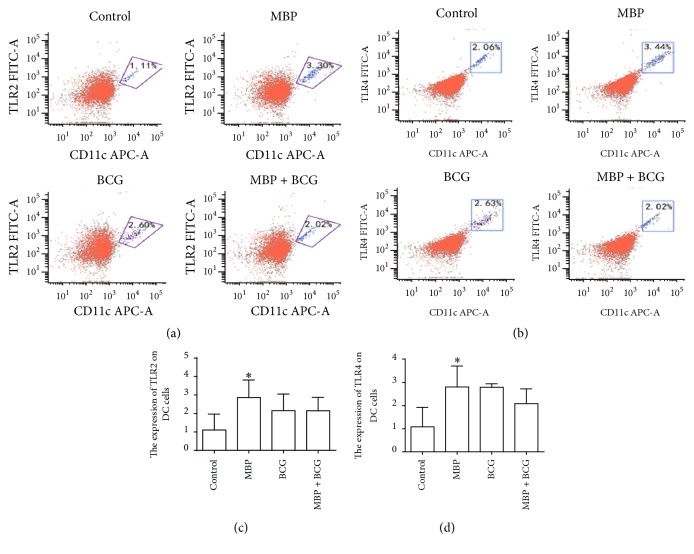
MBP upregulates the surface expression of TLR2/TLR4 on DCs. (a-b) TLR2/TLR4 surface molecule expression in DCs is shown in flow cytometry dot plots. (c-d) The percentage of CD11c^+^ cells expressing surface TLR2/TLR4 is shown in a data table. All data are expressed as the mean ± standard deviation from three independent experiments. ^*∗*^*P* < 0.05 compared with control group.

TLR signaling plays a critical role in the function of DCs, including phenotypic maturation and T cell stimulation. Much work has been completed to study the effects of TLRs on the maturation and function of dendritic cells [16–18]. In this study, we investigated the influence of MBP and BCG on TLR2/TLR4 expression in DCs from normal mice. The surface expression of TLR2 and TLR4 on DCs from normal mice stimulated with MBP, BCG, and MBP + BCG for 48 h was analyzed using flow cytometry. The data demonstrated that MBP significantly increased the percentage of CD11c^+^ cells expressing surface TLR2 from 1.11% in the control group to 3.30% in the MBP group. Similarly, MBP increased the expression of the TLR4 surface molecule on CD11c^+^ cells compared to the control group (3.44% versus 2.06%). BCG increased the surface TLR2 and TLR4 expression in DCs relative to the control group. The combination of MBP and BCG also increased the expression of the TLR2 surface marker but had very little effect on the expression of TLR4 in DCs (Figure 3).

### 3.4. TLR4 Signaling Is Required for the MBP Plus BCG-Induced Maturation of DCs

**Figure 4:**
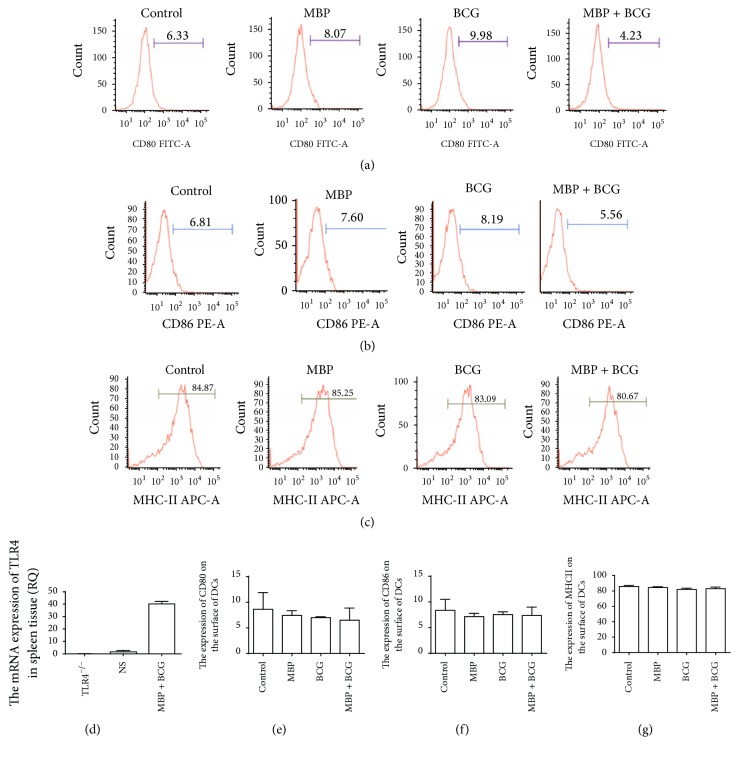
Effect of the combination of MBP and BCG on the phenotypic maturation of DCs from TLR4^−/−^ mice. Splenic DCs from TLR4^−/−^ mice (1 × 10^6^ cells/mL) were cultured with MBP, BCG, or the combination of MBP and BCG for 48 h and were analyzed using flow cytometry. (a–c) DCs collected from the different groups were stained with the following antibodies: FITC-conjugated anti-CD80, PE-conjugated anti-CD86, and APC-conjugated anti-MHC class II. The percentage of positive cells is shown in a flow cytometry histogram. (e–g) The percentage of CD11c^+^ cells expressing each surface molecule is expressed as the mean ± standard deviation and is shown in a bar graph. Data are derived from three independent experiments. ^*∗*^*P* < 0.05 is considered to indicate statistically significant differences, compared with control group derived from WT mice. (d) The mRNA level of TLR4 in spleen tissue from TLR4^−/−^ mice or from WT mice was measured with RT-PCR.

The mRNA level of TLR4 in spleen tissue from TLR4^−/−^ mice was verified using RT-PCR (real-time polymerase chain reaction) before completing these experiments. TLR4 was expressed in the spleens of WT mice but not in the spleens of TLR4^−/−^ mice (Figure 4(d)). The mRNA level of TLR4 in the spleens of WT mice was used as a positive control. To investigate the significance of TLR4 in the MBP plus BCG-induced upregulation of markers of a mature phenotype on DCs, DCs derived from TLR4^−/−^ mice were stimulated with MBP, BCG, or the combination of MBP and BCG for 48 h. Then, the expression of CD80, CD86, and MHC class II on the surface of DCs was analyzed using flow cytometry. Compared to DCs from WT mice, DCs from TLR4^−/−^ mice showed lower CD80 and CD86 expression (11.19% versus 6.33%; 15.62% versus 6.81%) (Figures 4(a), 4(b), 4(e), and 4(f)), indicating a more immature phenotype. TLR4 deficiency decreased the expression of the surface markers CD80 and CD86 on DCs; that is, the expression of the TLR4 molecule on the surface of DCs is very important to the maturation of DCs. No changes in the expression of MHC class II were observed in DCs from TLR4^−/−^ mice (Figures 4(c) and 4(g)). Moreover, MBP, BCG, and the combination of MBP and BCG failed to increase the expression of CD80, CD86, and MHC class II on the surface of splenic DCs from TLR4^−/−^ mice. These results show that the combination of MBP and BCG upregulates the expression of the surface markers CD80, CD86, and MHC class II in DCs via the TLR4 signaling pathway.

### 3.5. TLR2 Signaling Is Also Required for the MBP Plus BCG-Induced Maturation of DCs

**Figure 5:**
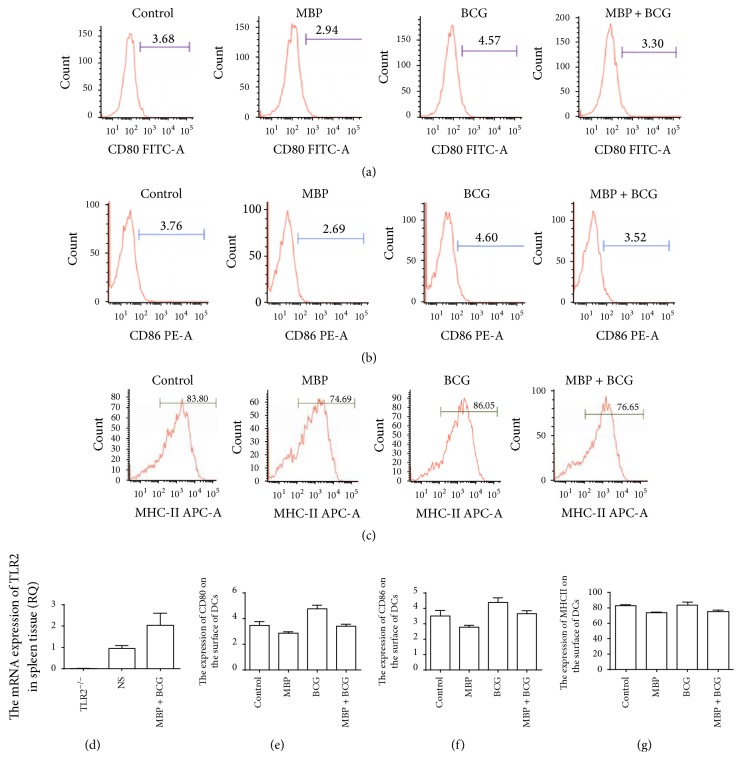
Effect of the combination of MBP and BCG on the phenotypic maturation of DCs from TLR2^−/−^ mice. Splenic DCs from TLR2^−/−^ mice (1 × 10^6^ cells/mL) were cultured with MBP, BCG, or the combination of MBP and BCG for 48 h and were analyzed using flow cytometry. (a–c) DCs collected from different groups were stained with the following antibodies: FITC-conjugated anti-CD80, PE-conjugated anti-CD86, and APC-conjugated anti-MHC class II. The percentage of positive cells is shown in a flow cytometry histogram. (e–g) The percentage of CD11C^+^ cells expressing each surface molecule is expressed as the mean ± standard deviation and is shown in a bar graph. Data are derived from three independent experiments. ^*∗*^*P* < 0.05 is considered to indicate statistically significant differences, compared with control group derived from WT mice. (d) The mRNA level of TLR2 in the spleens of WT mice or TLR2^−/−^ mice was detected with RT-PCR.

TLR2 mRNA expression was detected in the spleens of WT mice but not in the spleens of TLR2^−/−^ mice. The mRNA level of TLR2 in the spleens of WT mice was used as a positive control (Figure 5(d)). To further elucidate the functional significance of TLR2 expression on DCs, DCs isolated from TLR2^−/−^ mice were stimulated with MBP, BCG or the combination of MBP, and BCG for 48 h. Then, the expression of the maturation markers CD80, CD86, and MHC class II on the DCs was examined with flow cytometry. As shown in Figures 5(a), 5(b), 5(e), and 5(f), compared to DCs from WT mice, DCs from TLR2^−/−^ mice showed lower CD80 and CD86 expression (11.19% versus 3.68%; 15.62% versus 3.76%). DCs lacking TLR2 expressed lower levels of costimulatory molecules on their surface compared to DCs from normal mice. These results indicated that these cells had a more immature state. However, no changes were observed in MHC class II expression on DCs from TLR2^−/−^ mice (Figures 5(c) and 5(g)). Moreover, MBP, BCG, and the combination of MBP and BCG upregulated the expression of CD80, CD86, and MHC class II in DCs from normal mice, whereas these treatments failed to induce the upregulation of costimulatory molecules and MHC class II molecules in DCs from TLR2^−/−^ mice. These results showed that the combination of MBP and BCG upregulates the expression of the surface markers CD80, CD86, and MHC class II on DCs via the TLR2 signaling pathway.

### 3.6. The Effects of DC TLR2/TLR4 Expression on the Proliferation and Activation of CD4^+^ T Cells Induced by the Combination of MBP and BCG In Vitro

**Figure 6:**
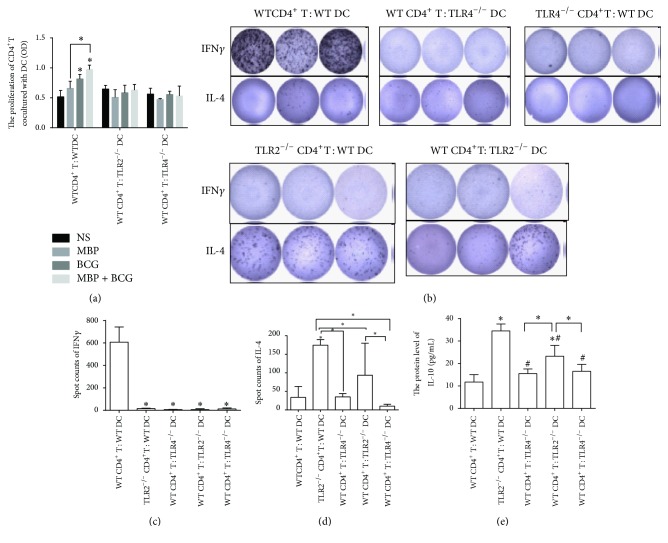
The direct and indirect effect of TLR2 and TLR4 on the production of cytokines in CD4^+^ T cells cocultured with DCs treated with the combination of MBP and BCG. (a) The function of TLR2/TLR4 expressed on DCs in the proliferation of CD4^+^ T cells from normal mice. CD4^+^ T cells from normal mice cocultured with DCs form TLR2^−/−^ mice or TLR4^−/−^ mice were stimulated with the combination of MBP and BCG for 48 h. The proliferation of CD4^+^ T cells was assayed with the CCK8 method. Data are expressed as the mean ± standard deviation and are shown in a bar graph. (b) Spots were counted by an ELISPOT reader. (c) ELISPOT cells showing IFN-*γ* production by CD4^+^ T cells cocultured with DCs from different groups after incubation with the combination of MBP and BCG. (d) ELISPOT cells showing IL-4 production by CD4^+^ T cells cocultured with DCs from different groups after incubation with the combination of MBP and BCG. (e) ELISA was used to detect the production of IL-10 in CD4^+^T cells cocultured with DCs from different groups treatment with the combination of MBP and BCG. ^*∗*^*P* < 0.05 is considered to indicate statistically significant differences compared with the coculture of CD4^+^ T cells from WT mice with DC cells from WT mice. ^#^*P* < 0.05 compared with coculture of CD4^+^T cells from TLR2^−/−^ mice with DC cells from WT mice.

From the results mentioned above, the combination of MBP and BCG synergistically increased the proliferation of CD4^+^ T cells from normal mice cocultured with DCs from normal mice relative to that observed in the control group. To detect the indirect effect of TLR2/TLR4 expression in DCs on the proliferation of CD4^+^ T cells, the proliferation of CD4^+^ T cells from WT mice cocultured with TLR2^−/−^ DCs or with TLR4^−/−^ DCs was assessed using a CCK8 assay following treatment with MBP, BCG, or the combination of MBP and BCG. As shown in Figure 6(a), the DCs lacking TLR2 or TLR4 failed to increase the proliferation of CD4^+^ T cells from WT mice after stimulation with MBP, BCG, or the combination of MBP and BCG. These results indicated that TLR2/TLR4 expression on DCs is very important to the proliferation of CD4^+^ T cells from WT mice stimulated with MBP, BCG, or the combination of MBP and BCG.

Moreover, to further detect the indirect effect of the expression of TLR2/TLR4 by DCs on the polarization of Th cells, the ELISPOT method was used to detect IFN-*γ* and IL-4 production by CD4^+^ T cells from WT mice cocultured with DCs from TLR2^−/−^ mice or TLR4^−/−^ mice that were treated with MBP, BCG, or the combination of MBP and BCG. The results showed that the combination of MBP and BCG failed to increase the level of IFN-*γ* produced by CD4^+^ T cells from WT mice cocultured with TLR2^−/−^ DC cells or TLR4^−/−^ DC cells. These results indicated that TLR2/TLR4 expression in DC cells plays an important role in polarizing the Th response toward Th1 (Figures 6(c) and 6(b)). The coculture of CD4^+^ T cells with DCs from TLR2^−/−^ mice or TLR4^−/−^ mice resulted in constitutively low amounts of cytokine production that remained unchanged after MBP or BCG stimulation (data not shown).

As shown in Figures 6(b)–6(d), compared to the coculture of CD4^+^ T cells from normal mice with DC cells from normal mice, the levels of IL-4 produced by TLR2^−/−^ CD4^+^ T cells cocultured with DCs from WT mice and CD4^+^ T cells from normal mice cocultured with TLR2^−/−^ DCs increased after stimulation with the combination of MBP and BCG. The increase in IL-4 production in cocultures of TLR2^−/−^ CD4^+^ T cells with normal DCs indicated that the combination of MBP and BCG has a direct influence on the activation of CD4^+^ T cells. Moreover, the data about IL-10 protein show the same tendency as compared with the results of IL-4 (Figure 6(e)). These results are in accordance with our previous observations [14]. This is also in line with previous studies showing that TLR2 engagement by CD4^+^ T cells enhances effector function and may contribute to protection against* Mycobacterium tuberculosis* infection [19]. In addition, no changes were observed in the level of IL-4 production in cocultures of TLR4^−/−^ CD4^+^ T cells with normal DCs and cocultures of normal CD4^+^ T cells with TLR4^−/−^ DCs after treatment with the combination of MBP and BCG (Figures 6(b)–6(d)).

### 3.7. The Combination of MBP and BCG Induced the Upregulation of TLR2/TLR4 Expression on DCs from WT Mice In Vivo

**Figure 7:**
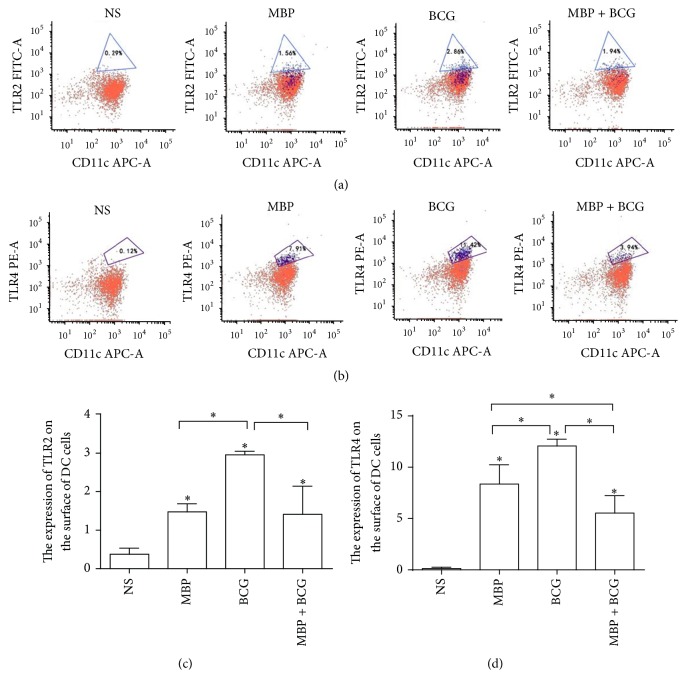
The effect of the combination of MBP and BCG on the expression of the surface molecules TLR2 and TLR4 on DCs in vivo. DCs isolated from the spleens of normal mice immunized with MBP, BCG, or the combination of MBP and BCG were incubated with FITC-conjugated anti-TLR4, PE-conjugated anti-TLR2, and APC-conjugated anti-CD11c at 4°C for 30 min. Then, cells were analyzed for the expression of TLR2/TLR4 on DCs with flow cytometry. (a) TLR4 expression on DCs from mice immunized with MBP, BCG, or the combination of MBP and BCG. (b) The expression of TLR2 on DCs isolated from the spleens of mice immunized with MBP, BCG, or the combination of MBP and BCG. (c-d) The percentage of CD11c^+^ cells expressing the TLR2/TLR4 molecules is expressed as the mean ± standard deviation and is shown in a bar graph. Data are derived from three independent experiments. ^*∗*^*P* < 0.05 is considered to indicate statistically significant differences compared with the NS group.

To detect the effect of the combination of MBP and BCG on the expression of the surface molecules TLR2/TLR4 on DCs in vivo, the expression of TLR2 and TLR4 on DCs from WT mice immunized with MBP, BCG, or the combination of MBP and BCG was analyzed using flow cytometry. Compared to the control group, TLR2 expression on the DCs from the MBP group, the BCG group or the MBP plus BCG group was significantly elevated (*P* < 0.05). The expression of TLR2 on DCs from the BCG group was higher than that in the MBP group and the MBP plus BCG group (*P* < 0.05). Compared with the control group, the expression of TLR4 on the surface of DCs from the MBP group, the BCG group and the MBP plus BCG group increased significantly (*P* < 0.05). The level of TLR4 on the DCs in the BCG group was significantly higher than that in the MBP group and in the MBP plus BCG group (*P* < 0.05) (Figure 7).

### 3.8. The Effect of TLR2 and TLR4 Expression on the MBP Plus BCG-Induced Expression of Costimulatory Molecules on DC Cells In Vivo

**Figure 8:**
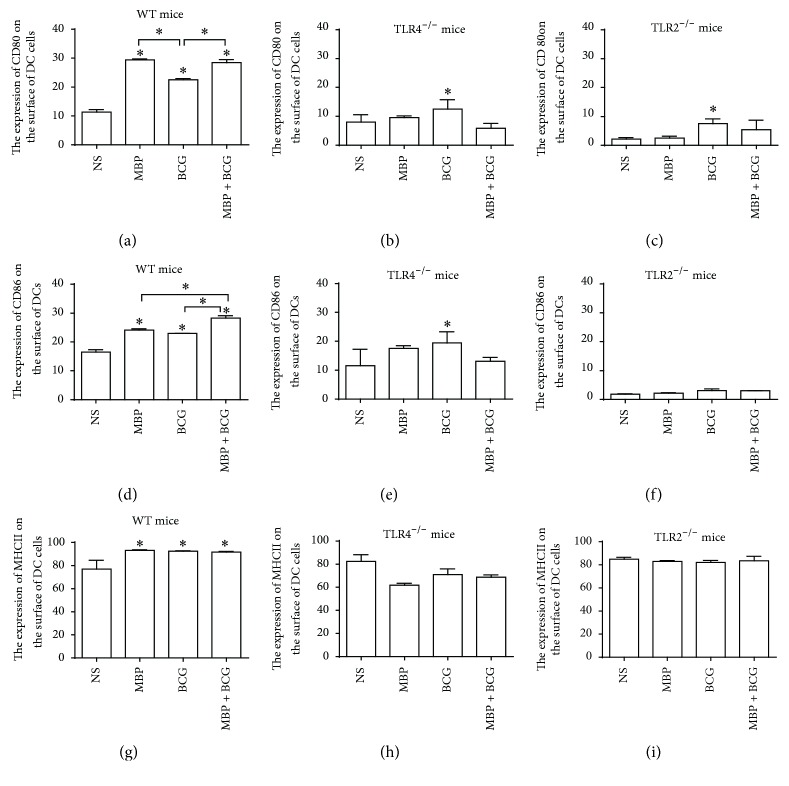
MBP plus BCG enhances CD80, CD86, and MHCII expression on DCs in vivo. DCs were isolated from WT mice ((a), (d), and (g)), TLR4^−/−^ mice ((b), (e), and (h)), or TLR2^−/−^ mice ((c), (f), and (i)) immunized with MBP, BCG, or the combination of MBP and BCG and were analyzed for CD80, CD86, and MHCII expression with flow cytometry. The percentage of CD11c^+^ cells expressing CD80, CD86, and MHCII is expressed as the mean ± standard deviation and is shown in a bar graph. Data are derived from three independent experiments. ^*∗*^*P* < 0.05 is considered to indicate statistically significant differences compared with the NS group derived from WT mice.

The in vitro experiments have demonstrated that MBP, BCG, and the combination of MBP and BCG play a role in promoting the expression of costimulatory molecules and in regulating the functional ability of DCs. To examine more directly whether the combination of MBP and BCG is very important in influencing the expression of costimulatory molecules on DCs through the TLR2/TLR4 pathways, an in vivo study further detected the influence of the combination of MBP and BCG on the expression of costimulatory molecules in DCs from WT mice, TLR2^−/−^ mice, and TLR4^−/−^ mice. A notable increase in CD80 and CD86 expression in DCs from WT mice was detected in the MBP, BCG, and MBP plus BCG groups relative to that in the control group (Figures 8(a) and 8(d)). A similar trend in MHC II expression on DCs was observed, although the difference did not reach statistical significance (Figure 8(g)). These results are in accordance with the in vitro experiments.

To test the contribution of the TLR4 molecule in the MBP plus BCG-induced functional ability of DCs, the expression of the CD80, CD86, and MHCII molecules on the surface of DCs from TLR4^−/−^ mice immunized with MBP, BCG, or the combination of MBP and BCG was examined with flow cytometry. TLR4 deficiency significantly decreased the expression of CD80, CD86, and MHCII on DCs. There was no obvious change in CD80, CD86, and MHCII expression in DC cells from TLR4^−/−^ mice immunized with MBP, BCG, or the combination of MBP and BCG (Figures 8(b), 8(e), and 8(h)). The expression of CD80 and CD86 on DCs from TLR4^−/−^ mice immunized with MBP, BCG, or the combination of MBP and BCG was significantly higher than that in TLR2^−/−^ mice (Figures 8(b), 8(e), 8(c), and 8(h)). These data suggested that the upregulation of costimulatory molecules in DCs induced by the combination of MBP and BCG may be more dependent on the TLR2 molecule than TLR4 molecule.

To confirm whether TLR2 molecules are involved in the functional ability of DCs induced by the combination of MBP and BCG, the expression of costimulatory molecules on DC cells derived from TLR2^−/−^ mice immunized with MBP, BCG, or the combination of MBP and BCG was analyzed by flow cytometry. As shown in Figures 8(c) and 8(f), TLR2 deficiency decreased significantly the expression of CD80 and CD86 on the surface of DC cells. The CD80 and CD86 expression in DC cells from TLR2^−/−^ mice immunized with MBP, BCG, or the combination of MBP and BCG also decreased significantly relative to the other groups derived from WT mice. There was no obvious change in MHC II expression in DC cells from TLR2^−/−^ mice immunized with MBP, BCG, or the combination of MBP and BCG (Figure 8(i)). This suggested that TLR2 molecules are very important in the expression of CD80 and CD86 on the surface of DC cells. These results indicated that the combination of MBP and BCG significantly increased the expression of CD80, CD86, and MHCII on the surface of DCs mainly through the TLR2 molecule.

### 3.9. The Effect of MBP and BCG on CD4^+^T Cell Activation via TLR2/TLR4 In Vivo

**Figure 9:**
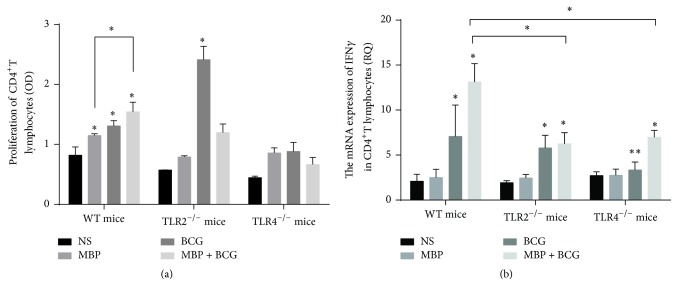
The effect of TLR2/TLR4 expression on the activation of CD4^+^ T cells induced by the combination of MBP and BCG in vivo. (a) CCK8 assays were used to analyze the proliferation of CD4^+^ T cells from different types of mice immunized with MBP, BCG, or the combination of MBP and BCG. (b) CD4^+^ T cells were isolated from WT mice, TLR2^−/−^ mice, or TLR4^−/−^ mice immunized with MBP, BCG, or the combination of MBP and BCG, respectively. The mRNA levels of IFN-*γ* in the CD4^+^ T cells from these groups were measured with RT-PCR. ^*∗*^*P* < 0.05 is considered to indicate statistically significant differences compared with the NS group derived from WT mice. ^*∗∗*^*P* < 0.05, compared with BCG group from different types of mice.

To confirm the functional involvement of the combination of MBP and BCG in the proliferation of CD4^+^ T cells, we next examined the proliferation of CD4^+^ T cells from WT mice, TLR2^−/−^ mice, and TLR4^−/−^ mice immunized with MBP, BCG, or the combination of MBP and BCG. The proliferation of CD4^+^ T cells was analyzed with CCK8 kits. As shown in Figure 9(a), the proliferation of CD4^+^ T cells from normal mice immunized with the combination of MBP and BCG was significantly higher than that in NS, MBP, and BCG groups. These data indicated that the combination of MBP and BCG promotes the proliferation of CD4^+^ T cells in vivo. To observe whether TLR2 and TLR4 molecules influence the proliferation of CD4^+^ T cells, TLR2^−/−^ mice or TLR4^−/−^ mice were immunized with MBP, BCG, or the combination of MBP and BCG, respectively. Then, CD4^+^ T cells were isolated from different groups and the proliferation was detected using CCK8 kits. In contrast to normal mice, MBP and BCG failed to increase the proliferation of CD4^+^ T cells from TLR2^−/−^ mice or TLR4^−/−^ mice in vivo. In this study, these results suggested that the proliferation of CD4^+^ T cells induced by the combination of MBP and BCG may be dependent on TLR2/TLR4 in vivo.

To further confirm the effect of the combination of MBP and BCG on the Th1 response in vivo, we examined the level of IFN-*γ* mRNA in CD4^+^ T cells from mice in different groups, including MBP group, BCG group, and the combination of MBP and BCG group. As shown in Figure 9(b), CD4^+^ T cells from normal mice immunized with BCG expressed significantly higher levels of IFN-*γ* mRNA than CD4^+^ T cells in NS group or MBP group. The combination of MBP and BCG significantly increased the mRNA levels of IFN-*γ* in CD4^+^ T cells relative to those in the control group, MBP group, and BCG group (*P* < 0.05). However, the level of IFN-*γ* mRNA in CD4^+^ T cells from TLR4^−/−^ mice or TLR2^−/−^ mice significantly decreased, compared to the combination of MBP and BCG immunized normal mice. In addition, lack of TLR4 decreased the mRNA level of IFN-*γ* in CD4^+^ T cells following immunization with BCG. No change was observed in the IFN-*γ* mRNA level in CD4^+^ T cells from TLR2^−/−^ mice immunized with BCG. This indicated that the combination of MBP and BCG plays an important role in the polarization of Th cells toward a Th1 phenotype partly through TLR2/TLR4 expressed in CD4^+^ T cells in vivo.

## 4. Discussion

It is well known that DCs are an important component of innate immunity and that these cells play a critical role in bridging innate and adaptive immunity by modulating the function of T cells [20, 21]. In this study, we demonstrated that the combination of MBP and BCG plays an important role in the maturation and activation of DCs and promotes the Th1 response through the expression of TLR2/TLR4 molecules. The experiments in vitro found that the combination of MBP and BCG promoted the expression of costimulatory molecules on DCs and increased the secretion of IL-12 protein, which induced the activation and maturation of DCs. The upregulation of surface costimulatory molecules on DCs is critically important for the modulatory effect of antigen presentation on the function and development of T cells [22]. In addition, we demonstrated that the combination of MBP and BCG enhanced the proliferation of CD4^+^ T cells cocultured with DC cells from WT mice and increased IFN-*γ* production by CD4^+^ T cells cocultured with DC cells from WT mice. These results indicated that the combination of MBP and BCG induces the maturation and activation of DCs, indirectly promotes the proliferation of CD4^+^ T cells, and induces CD4^+^T cell polarization toward a Th1 phenotype.

TLRs are one of the most extensively studied PRR families, and the activation of TLRs plays a critical role in the innate response and in directing acquired immunity [23–25]. In addition, the expression of TLRs on antigen presenting cells (APC), mainly on dendritic cells, leads to the maturation of DCs and induces the priming of naive T cells to drive adaptive immunity [26, 27]. The activation of TLRs results in the upregulation of costimulatory molecule expression on the surface of DCs, including CD80 and CD86. The costimulatory molecules CD80 and CD86 interact with CD28 on naive CD4^+^ T cells to enhance APC activity. Major histocompatibility complex II on DCs mainly presents antigen peptides to the T cell receptor (TCR). The activation and maturation induced by the recognition of pathogen-associated patterns via TLRs provide signals to naive T cells and prime naive T cells toward specific T helper profiles [28, 29].

In a recent study, the major components of* Antrodia cinnamomea* promoted proinflammatory cytokine production by DCs and the maturation of DCs via the TLR2/TLR4 and NF-KB/MAPK signaling pathways [30]. We demonstrated that the combination of MBP and BCG upregulates the expression of TLR2 and TLR4 on the surface of DCs. We hypothesized that the DC maturation induced by the combination of MBP and BCG occurred via the TLR2/TLR4 molecules expressed on DCs. To confirm the molecular mechanism, DCs from TLR2^−/−^ mice or TLR4^−/−^ mice were stimulated with MBP, BCG, and the combination of MBP and BCG in vitro, and the expression of CD80, CD86, and MHCII on the surface of these DC cells was analyzed with flow cytometry. The data indicated that TLR2-deficient DCs showed lower expression of CD80, CD86, and MHC II than DCs from normal mice. Moreover, stimulation with MBP and BCG did not increase the expression of costimulatory molecules on DCs. TLR4 deficiency also decreased the expression of CD80, CD86, and MHC II on DCs despite stimulation with MBP, BCG, or the combination of MBP and BCG. Thus, our results clearly demonstrate that TLR2 and TLR4 are required for the stimulatory effect of the combination of MBP and BCG on the function of DCs.

The in vitro experiments studied the effect of DCs from TLR2^−/−^ mice or TLR4^−/−^ mice on the proliferation of MBP plus BCG-induced activation of Th1 cells. The data indicated that the proliferation of CD4^+^ T cells did not increase in response to coculture with TLR2^−/−^ DCs or TLR4^−/−^ DCs treated with the combination of MBP and BCG. Lack of TLR2 or TLR4 molecule in DCs decreased the production of IFN-*γ* and IL-4 of CD4^+^ T cells induced by the combination of MBP and BCG. It indicated that DCs play an indirect role in the polarization of CD4^+^T cells induced by MBP and BCG via TLR2/TLR4. In addition, the secretion of IFN-*γ* was very low in CD4^+^ T cells from TLR2^−/−^ mice cocultured with DCs from WT mice after stimulation with MBP and BCG, but the secretion of IL-4 was very high. The data showed that TLR2 expressed by CD4^+^ T cells directly promoted CD4^+^ T cell polarization toward the Th1 phenotype. The level of IFN-*γ* production and IL-4 production were very low in CD4^+^ T cells from TLR4^−/−^ mice cocultured with DCs from WT mice. This suggested that the expression of TLR4 on the surface of CD4^+^ T cells directly promoted the polarization of CD4^+^ T cells toward Th1 and Th2 phenotype after stimulation with MBP and BCG. The results of the present study are consistent with our previous study that MBP and BCG directly induced the polarization of CD4^+^ T cells toward a Th1 phenotype and that the molecular mechanism is associated with the TLR2/TLR4/TLR9 signaling pathways [14].

The aim of study in vivo was to investigate the effect of the combination of MBP and BCG on the maturation and antigen presenting function of DCs. The combination of MBP and BCG promoted the upregulation of TLR2/TLR4 expression and the expression of costimulatory molecules on DCs. The results of study in vivo further confirm with the in vitro results. Moreover, the combination of MBP and BCG significantly increased the proliferation and IFN-*γ* mRNA level of CD4^+^T cells from normal mice, but lack of TLR2 or TLR4 has a blocked effect on the proliferation and IFN-*γ* mRNA level of CD4^+^T cells in vivo study. The results suggested that the combination of MBP and BCG promotes the activation and maturation of DC and has an indirect effect on the polarization of CD4^+^ T cells toward Th1 response. Moreover, the molecular mechanism may be associated with the TLR2/TLR4 signaling pathways.

In summary, the present study mainly explored the expression of costimulatory molecules and the activation of DCs induced by the combination of MBP and BCG in vivo and in vitro. The results suggested that the expression of TLR2/TLR4 may be one of the potent mechanisms involved in altering DC maturation and in priming a Th1 response after stimulation with the combination of MBP and BCG. The combination of MBP and BCG has the potential to be used as a TLR2/TLR4 agonist or as an adjuvant for enhancing immune responses in immune therapies.
